# Sustainable metals: integrating science and systems approaches

**DOI:** 10.1098/rsta.2023.0247

**Published:** 2024-11-04

**Authors:** Julian M. Allwood, Dierk Raabe

**Affiliations:** ^1^ Department of Engineering, University of Cambridge, Trumpington Street, Cambridge CB2 1PZ, UK; ^2^ Max-Planck-Institute for Sustainable Materials, Düsseldorf 40237, Germany

**Keywords:** sustainability, metals, interdisciplinarity

## Abstract

This article introduces a special issue of the transactions arising from a Royal Society Discussion Meeting on ‘sustainable metals’. Recognizing that progress to date toward the goals of ‘sustainability’ has been limited, the meeting aimed to open up a new level of interdisciplinary dialogue, collaboration and discussion of disruptive approaches. In this paper, the major concerns of sustainability are enumerated, and climate change is identified as the most urgent. The constraints on deploying technical innovations at scale and speed are discussed, suggesting that much of the required change will require using existing technologies differently, and many opportunities of this type have been overlooked. These constraints also give useful direction for future research and suggest an expanded future role for scientists. Previously, scientists and technologists have aimed largely to ‘solve’ problems in sustainability through invention. This introductory paper argues that they have an equally important role as participants in the complex societal discussions required to identify pathways to change. Scientific expertise is as important for explaining what cannot be achieved in time or at scale, as it is for promoting the excitement of invention.

This article is part of the discussion meeting issue ‘Sustainable metals: science and systems’.

## 1. Introduction

In proposing this Discussion Meeting and Special Issue to the Royal Society, we aimed to bring together insights into sustainable metals from our two surprisingly separated backgrounds. From systems engineering, we recognize that in the next few decades, emissions-free primary production of metal will be less than total demand, which motivates the search for innovation in technology, business and policy to live well with less new metal [1]. Recognizing this gap between future supply and demand has, for example, helped us develop process innovations to reduce by two-thirds the sheet metal scrapped when making automotive ‘bodies-in-white’ [2] and to identify the first electric route for cement recycling [3]. From materials science, we recognize that metallurgy impacts global sustainability both directly (through the environmental and social consequences of production) and indirectly (through the benefits of using metals to deliver new functions) [4]. The primary concern of this meeting is the direct impacts, which have been under-prioritized by materials scientists focused on novel compositions. However, there are rich opportunities for innovation in tackling such direct impacts, for example, with new reductants or electrochemical process routes, although all such innovations are subject to unavoidable thermodynamic and kinetic constraints.

The purpose of the Discussion Meeting and this issue is to stimulate new connections, not just between our own disciplines but across the spectrum of skills involved in shaping the future metals industry. The papers in this volume demonstrate the scope for new collaborative approaches, and in this introduction, we aim to set the scene both for the meeting and for future work in this vital area.

## 2. The meaning of ‘sustainability’

The word ‘sustainable’ in the sense we are using it here was defined in 1987 in the Brundtland Report [5] as the aim of ‘development that meets the needs of the present without compromising the ability of future generations to meet their own needs’. For at least two decades after this definition was established, ‘sustainability’ was linked to metrics of the ‘triple-bottom-line’ of environmental, social and economic progress.

Table 1 presents the main concerns of such triple-bottom-line approaches, assembled from the environmental impact categories of Life Cycle Analysis [6] and the widely discussed Sustainable Development Goals of the United Nations [7].

**Table 1.** The concerns of ‘sustainability’ expressed in the goals of the ‘triple-bottom-line’.

environmental	social	economic
climate change	no poverty	affordable clean energy
ozone depletion	zero hunger	economic growth
acidification	good health and well-being	industry, innovation and infrastructure
eutrophication	quality education	
resource depletion	clean water and sanitation	
human toxicity	reduced inequalities	
eco-toxicity	sustainable cities and communities	
water use	responsible consumption and production	
land use	decent work	
ionizing radiation	peace, justice and strong institutions	
particulate emissions		
species diversity		

It is immediately apparent that table 1 sets a challenging and even impossible agenda. It is difficult to think of interventions that make meaningful progress on some or all of these indicators without detriment to any others. This is particularly the case for the economic criteria in the table, which reflect ‘business as usual’ as if that was as important as environmental and social concern. The idea of the triple-bottom-line has, therefore, failed to initiate significant change and has even been used as an excuse for ‘green washing’. Reluctant managers use this framing to claim steady progress with negligible changes while opposing interventions that would lead to significant environmental or societal progress but might reduce profits.

This is hardly surprising. Following the principles of the free market, corporations aim to return maximum value to their shareholders subject to the rules within which they are allowed to operate. If there were profitable interventions that also had environmental and societal benefits, it would have been irrational for corporations not to adopt them already.

It is, therefore, very likely that the real pursuit of environmental and societal goals requires changes to the constraints within which corporations may operate. This approach is familiar from taxes on processing or storing wastes and has been a stimulus for new science, for example, in reprocessing the red mud of alumina extraction [8]. Such taxes are the basis of three decades of academic discussion about ‘pricing carbon’ as a means to motivate corporations to reduce global emissions [9], although this has proved politically unrealizable [10]. Instead, the mechanism by which corporations can be persuaded to change their processes is through health and safety legislation. This is familiar in food and drug safety, and is how we ensure product safety, in applications from bridges and cars to domestic appliances. Regulation is the means by which most environmental and societal change is delivered, and as it changes the rules within which companies can operate, it overides many of the concerns in table 1 about the profitability of existing corporations operating under existing rules.

However, the remaining concerns in the table cover so much breadth that it is difficult to make progress on them all at equal speed. The table must, therefore, be interpreted with some form of priority, and this depends on how rapidly the concerns of table 1 might become existential threats to humans.

Some of the environmental impacts in table 1 are fundamentally local: toxic releases to water, for example, affect single rivers at a time, and historically, this problem has been addressed when a sufficiently vocal middle-class lobby has petitioned the government to regulate the polluter. Some of the social indicators, such as ‘good health and well-being’, are fundamentally global yet probably unreachable. Meanwhile, global peace has been a universally recognized target from the beginning of civilization, but one that has never been achieved for a sustained period. And despite the rhetoric of developed economies, inequality is something that motivates little action [11]. According to United Nations reports, one in 11 people alive today (and one in five in Africa) are hungry [12], yet in the UK, on average, we consistently spend twice as much on our pets as we give to all charities whether targeting hunger or any other cause [13].

Species loss is undesirable, but it is difficult to put a timeline on the harms it creates. Commentators often discuss the risks of losing pollinators, but our cereals are wind-pollinated, and it is likely that we could compensate with alternative approaches without existential risk to our own species. It is therefore probably important but not urgent.

Lastly, where resource depletion has occurred, our response to date has been to compensate by using more energy—for example, in extracting metals from less pure ores or in creating new freshwater by energy-intensive desalination of seawater. The problem of resource depletion, therefore, quickly turns into a problem of energy supply, which in turn becomes a problem of climate change.

Climate change is the most urgent and most global priority of table 1. According to Working Group Two of the Intergovernmental Panel on Climate Change (IPCC), a temperature rise above 2°C will lead to a ‘very high probability of severe impacts’. They predict that continued global inaction on global warming (despite political rhetoric, global emissions have risen by 50% since the United Nations Framework Convention on Climate Change was signed in 1992) will lead to temperature rises of 2.5–4.5°C by the end of this century [14]. And since the IPCC’s last report, global average temperatures have been above their most pessimistic forecast every year.

Climate change is, therefore, an immediate existential threat to the human species and requires action faster than any other goal in table 1. As a result, a practical definition of ‘sustainability’ to motivate action in the metals industry is to cut global emissions to zero by mid-century while at least not worsening global inequality or species diversity.

This pragmatic assessment has far-reaching consequences. Failure to act on climate change will lead to problems with food supply, and eventually a global war over food access. This will cause devastating harm, particularly in developing countries at the equator, whose agriculture will be most affected and who have the least chance to buy their way out of trouble. Acting on climate change may well challenge conventional ideas of economic development and may require some economic contraction in the developed world. However, given the urgency and scale of action to avoid devastating harm, the question is not ‘how do we have everything we want, while also living sustainably?’ Instead, research on sustainability topics should be motivated by asking, ‘within the environmental budgets that allow us a sustainable future, how can we live best?’

## 3. Urgency and resource constraints

**Figure 1.**
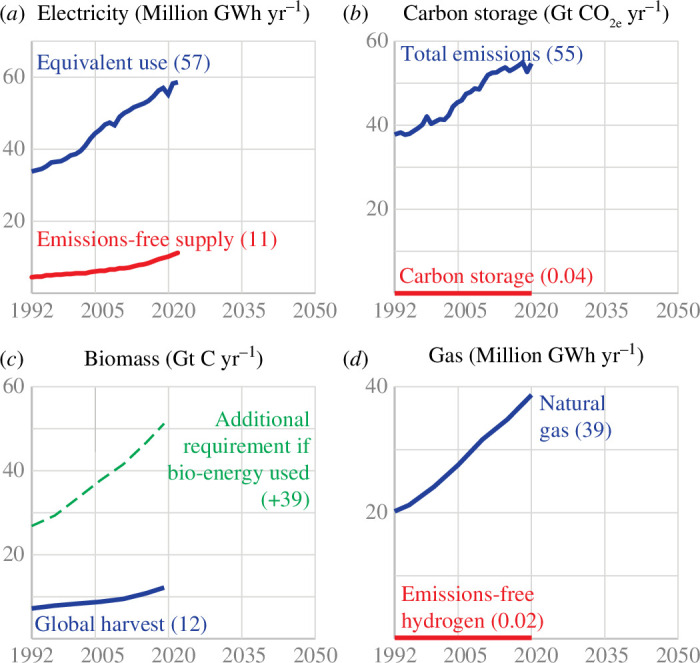
Fundamental resources available for climate mitigation at a global scale: (*a*) emissions-free electricity compared to total demand if all energy uses electrified, (*b*) carbon storage compared to global emissions and (*c*) total global biomass harvest for fuel, structure, food and fodder and compared with the additional harvest required if all electricity generation and vehicles were powered by biomass. As a consequence, (*d*) global emissions-free hydrogen production is negligible and unlikely to grow due to the constraints in (*a*) and (*b*). Figure adapted from [15].

Resource constraints and rates of deployment and scalability have largely been overlooked in discussions about sustainability, but nevertheless, they will limit what can be delivered in the few decades we have left to act. Recognizing these constraints is an essential basis for useful science on sustainable metals. Figure 1 illustrates the problem. All approaches to climate mitigation depend fundamentally on three basic resources: emissions-free electricity, carbon storage and biomass. (There are currently many more demonstrations of carbon capture than storage, and despite discussion of carbon dioxide utilization, no significant applications have scaled to date, so the fundamental constraint is carbon storage.)

Figure 1*a* shows that global emissions-free electricity generation has always previously grown at a linear rate, not exponentially, as might happen for a new model of mobile phone. Emissions-free generation currently delivers approximately a fifth of the supply we would require if all energy uses were electrified.

Figure 1*b* shows that carbon storage, discussed in policy as if it was an immediately available option, has been deployed at such small scale that global capacity is currently 0.04% of annual emissions and has grown at a rate of just 0.008% per year for the past decade. Any reasonable interpretation of this graph compared to the timeline on which climate action is required leads to the conclusion that carbon storage will not be available at scale in the time we must act.

Meanwhile, figure 1*c* demonstrates that biomass cannot contribute significantly to future energy supplies, because it has low energy density, so too much new harvest would be required to reach meaningful scale.

Techno-optimists, whose rhetoric dominates corporate and political discussion of climate change, respond to the first three graphs of figure 1 with the remark that ‘we will just have to go a bit faster’. However, that is extremely difficult to achieve. The large energy infrastructure projects of installing new generation or new storage projects require public finance, which must be traded off with other priorities such as health or education. They also require public consent at local and national scales to address concerns about location, land rights, access, safety, legal compliance, environmental and community impacts. Once this is agreed, contracts must be negotiated and the complex projects of construction and commissioning can begin. All of these steps take a long time and they cannot easily be accelerated in democracies [16,17].

A critical consequence of these resource constraints illustrated in figure 1*d* is that it is unlikely that manufactured fuels such as ammonia or, in particular, hydrogen will be available at significant scales in the next few decades. This is important and challenging for metallurgists. Hydrogen has numerous advantages as a fuel and a reductant, not least that the product of its combustion is water. However, figure 1*d* demonstrates that the current global production of emissions-free hydrogen is negligible. Without a massive supply of carbon storage or surplus emissions-free electricity, neither of which will be available soon, emissions-free hydrogen supplies can not grow substantially over the time we have to act on climate change. As a result, emissions-free hydrogen will make little if any contribution to replacing the world’s current high-emitting metal production capacity, which makes around two billion tonnes of liquid metal per year.

In parallel, as the shift from fossil to renewable energy supply will be material-intensive, some of the limited future supply of resources must be shifted from the operation of equipment to producing the materials required to construct equipment that can operate without emissions. The trade-off between the impacts of constructing new equipment and the benefits of its use is at the heart of a systems view of sustainability.

## 4. The role of science in the pursuit of sustainability

For many scientists, their role should be the uninhibited pursuit of discovery for its own sake. Many benefits we enjoy today are unanticipated consequences of past science, so this view is well-founded. Discovery-led science, like literature, musical composition and the plastic arts, will always continue because the people who are best at it, cannot avoid doing it.

However, it is optimistic to hope that undirected discovery-led science will turn up responses to global warming that can be scaled globally at sufficient speed to reduce greenhouse gas emissions to safe levels. For example, despite the obvious attractions that such discoveries might have, there have been no significant breakthroughs promising step changes in energy storage or generation in the past 30 years.

At first sight, this is disappointing. However, the technologies that cause global pollution problems today, from fossil combustion to metallurgy and cement, have developed over centuries. Huge investments and industries stand behind them, and the managers of incumbent production firms have a strong motivation to protect the status quo. Science must always be given a chance to challenge incumbent solutions, as for example, shown by the invention of ammonia as fertilizer or batteries to store energy. However, as these examples show, even such attractive inventions do not scale overnight.

This discussion is vital to the context of this special issue and to the politics of science funding. Science needs creative space, without which, disruptive changes are unlikely to happen. However, scaling up even hugely advantageous disruptions will always take time. Science funding bodies could helpfully reflect this reality: if science funding is linked to the delivery of reaching zero or near-zero emissions within the next two to three decades, the focus must be on modifications to existing technologies that can scale rapidly. It is very likely that such solutions will also require societal restraint as it will be impossible to substitute all current emitting activities in such a short time. In contrast, if science funding is linked to expanding a new zero-emissions economy, one that can grow once emissions have been reduced to a safe level, the requirement for urgency is removed, and discovery-led science can explore the expansion of human well-being across the globe, in a world without emissions.

To contribute to the goal of sustainability, science must then be targeted, focusing on the right problems while recognizing resource constraints and scale.

The habits of discovery-led science have led to a narrowing in the focus of materials science over the past generation. Where in the 1960s, textbooks of material science covered the whole supply chain of material production and use including its role in society [18], the recent focus of materials science has been innovation in composition and properties. This may lead to some of the ‘indirect’ benefits mentioned above but has drawn attention away from the direct impacts of metals production. Instead, the goal of sustainability requires an urgent focus on the processes by which metals are produced from ores or recycled, including innovations that lead to a reduction in the variety of alloys in use and facilitate higher quality upcycling of used metal. This requires a shift of focus away from novel (and often less sustainable) material compositions and properties toward disruptive and scalable innovations in processing that reduce the emissions, waste and energy of primary synthesis and upcycle used materials efficiently. Where product lifetimes are limited by material degradation, existing materials must be made more robust against environmental or operational decay, be it through better material protection against corrosion and hydrogen attack, batteries with lower capacity loss over time or catalysts that can prevail in contaminated redox environments.

As a result, science can contribute to the goals of sustainability if it is focused and intimately connected to the realities of resource constraints and scalability, and this requires collaboration.

**Figure 2.**
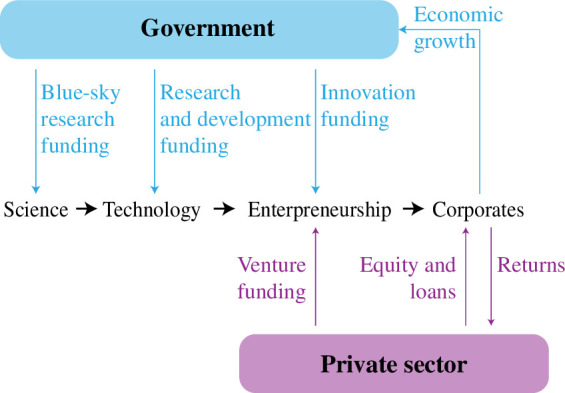
Simplified representation of how governments expect to see returns on investments in science funding.

Figure 2 illustrates the conventional model by which science contributes to economic well-being. In the virtuous circle implied by this model, public funding is justified for science on the grounds that it creates innovations that can be protected and commercialized, through a mix of public and private finance, creating new economic growth, which returns greater public funds for future use.

However, behind this model lies the assumption that science-led innovation will attract new spending from willing customers in return for new end-user benefits. The discussion of table 1 above demonstrated that the goals of sustainability require attention, precisely because they do not emerge from the normal practice of private sector commerce under today’s rules. If there were customers willing to pay more for ‘more sustainable’ products, the private sector would already have met their demand.

Instead, the innovations that lead to the real delivery of sustainability must largely lead to different ways to deliver familiar ends. Endless discussion of co-benefits in sustainability has looked for means to find different benefits from sustainable innovations [19], but despite a few examples (such as the benefit of improved air quality from using electric cars), it is unlikely that many inventions compatible with a zero-emissions future will offer previously unavailable customer benefits. In some cases, such as the high-price vehicle market, customers are beginning to show willingness to pay a ‘green premium’ for products with reduced operational or embodied emissions, and this is a powerful enabler of change [20]. However, in general, for customers without this motivation, the interventions that will reduce emissions significantly are unlikely to lead to new conventional benefits. If reducing emissions requires investment to change a production process, the model of figure 2 no longer creates the right motivations, which must instead be provided by regulatory change.

## 5. An expanded requirement for interdisciplinarity

This reality places a new emphasis on interdisciplinary collaboration that goes far beyond the connections of previously separated sciences. If the delivery of sustainability at speed and scale challenges existing market dynamics, it can only be brought about by an informed and wide-ranging public discussion that evaluates the feasibility and costs of different options and timescales against the costs of inaction and the balance of other priorities.

For many years, scientists working on sustainability have seen it as an additional motivation for what they were going to do anyway. (This is illustrated with extreme irony in the surprisingly rich literature on ‘sustainable bullets’ [21]). Similarly, corporate and political leaders promoting sustainability have done so only subject to the precondition that the organization that they lead prospers as a result of the actions they support.

This is not enough. Action on climate change, in particular, and on most of the other urgent priorities listed in table 1, will challenge some existing institutions. In some cases, for example, for the oil and gas sector, companies which have failed to construct carbon storage at scale despite record profits in recent years, meaningful action requires closure of most if not all of their existing assets.

Meaningful work on sustainability must place environmental and societal goals ahead of assumptions about what the solution might entail.

The cost of failure to address climate change is so severe that there can be no presumption about what we ‘must have’ as part of the solution. For example, for two generations, the habit of flying in fossil fuel-powered aeroplanes has grown rapidly, quadrupling since we signed the United Nations Framework Convention on Climate Change, and still untaxed due to the 1944 Chicago convention. The goal of having zero emissions by 2050 requires that all existing fossil aeroplanes are taken out of service, and because of the resource constraints illustrated in figure 1, there will be few if any alternatives available by then. As a result, almost all flights must end to reach zero emissions in the short term, yet politicians and the negotiators at the United Nations ‘Conference of the Parties’ (COP) meetings do not even mention the emissions of flying.

To date, climate policy has been mired in an unreality. Analysts forecast future demand assuming the precondition of economic growth as usual as if climate change did not exist. They then ‘back-cast’ the requirement for resources to supply this demand, as if stating a requirement means that it can be met. This illusion is supported in many cases by scientists overstating the speed at which future discoveries might reach a global scale. It has allowed political negotiators at the COP meetings to assume a portfolio of solutions that would require eight times more emissions-free electricity and 600 times more carbon storage than we actually have [22]. This cannot be delivered in the time available. It is fantasy, not science.

The real pursuit of sustainability and the science that supports it must instead start by forecasting the lowest risk estimate of the resources likely to be available to us. From this, we can back-cast options to use these limited resources, using innovations to improve the benefit we receive, rather than shifting the responsibility for delivery to other unknown hands.

Progress in sustainability to date has been held back woefully by scientists failing to apply the rigour of their usual methods to the realities of delivering change at speed and scale. It is reasonable for scientists to recognize that this is not their expertise, but only if they then form connections to ensure that their rigorous insights are connected to the wider societal dialogue about change.

**Figure 3.**
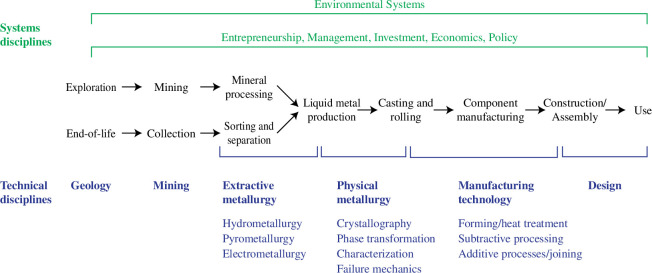
An illustration of the range of disciplines required to collaborate effectively to bring about a rapid systems transformation in the global supply of metals.

Figure 3 attempts to demonstrate the wider network of disciplinary approaches required to implement such meaningful change in reality. It is no longer adequate for science and technology to expect to ‘throw solutions over the wall’ to ‘a system’ that will deliver implementation at scale. Scientists must instead be part of the conversation about implementation.

Instead of overstating the potential of future discoveries, scientists must engage in new and humbler collaboration, with a much wider range of collocutors, including not just engineers and commercial partners but economists, policy analysts and public debate. Change with the required speed and scale will probably start locally and spread by copying good examples from elsewhere, so by connecting science within the whole system of change, including the journey to delivery at a meaningful scale, scientists will be able to embrace a new role for themselves. If they do so, they could accelerate the actions, including restraint, that will be essential to delivering a sustainable climate.

## 6. Open questions

With two to three decades remaining to transform global economies to live as well as possible with no emissions, we can anticipate some key features of the interventions that matter. Probably, most scalable solutions will involve material substitutions, rather than new materials, with changes to equipment configuration, tooling or control, rather than entirely new processes.

Out of the discussions at the Royal Society meeting leading to this issue, we can offer a first list of open questions in the hope that it stimulates new activity and interest in this vital area.

— Most *recycling* today is downcycling. By what process or system innovations can recycling return metals with higher compositional control, and what is the balance between energy input and metallic purity? Which elements can be separated from which others, at what cost and by which process? How do residual elements (in recycled metals) affect processing, microstructure and properties?
— During the transition to zero emissions, *primary* extraction of metals from ores will continue for some time, so what interventions can most effectively reduce the impact of existing equipment such as blast furnaces for making pig iron or non-ferrous metal production from minerals with low and reducing metal content?
— What are the potentials for *re-mining* the tailings, slags or wastes of earlier eras?
— For which products and processes can we *replace* the use of carbon- and energy-intense alloys such as in hard magnets containing rare-earth elements?
— What equipment modifications can be delivered within the limited *capital* budgets available in the metals sector?
— In addition to emissions-free electricity, carbon storage and biomass noted in figure 1, which other *resources* (e.g. sulphates or critical alloying elements) or by-products (such as sulphuric acid) will constrain future metals production or process innovations?
— How can metals scientists best support *public discussions* of regulation, technological potential, product design and environmental priorities including the limits to innovation and scale?
— How can we deliver the most service from a *limited metal supply* in the rapid transition to a zero-emissions world, and are there ‘low material intensity’ pathways to economic development?
— Which material *substitutions* (alloy-for-alloy, metal-for-metal and non-metal-for-metal) in processing or product design can lead to the most effective reductions in environmental harm?
— How are process efficiencies and costs, social impacts, flexibility and system efficiencies influenced by the *scale* of operations?
— What *trade-offs* matter between the environmental impact of metals production and its use in different applications, and how should these be prioritized?
— How can we translate these considerations of a more holistic, cross-disciplinary and less comfort zone-oriented view at this challenge into *education*, in basic natural sciences, engineering, humanities and business?

The 14 papers of this issue aim to begin addressing some of these questions and are introduced in table 2.

**Table 2.** The papers in the issue.

theme	lead author	title
environmental systems	Takuma Watari	Decarbonizing the global steel industry in a resource-constrained future—a systems perspective [23]
systems and operations	Claire Davis	Reuse, remanufacturing and recycling in the steel sector [24]
systems and manufacturing	Julian M. Allwood	Material efficiency at the component level: how much metal can we do without? [25]
systems and construction	Cyrille Dunant	What is the embodied CO_2_ cost of getting building design wrong? [26]
application innovation	Laurine Choisez	Influence of impurities on the use of Fe-based powder as sustainable fuel [27]
application innovation	Ziyuan Rao	Active learning strategies for the design of sustainable alloys [28]
process innovation	Katrin Daehn	A key feedback loop: building electricity infrastructure and electrifying metals production [29]
process innovation	Dierk Raabe	Sustainable metals: integrating science and systems approaches (this paper)
process innovation	Kivanc Korkmaz	System analysis with life cycle assessment for NiMH battery recycling [30]
mining	Eva Gerold	Sustainable extraction and recycling of non-ferrous metals: a review from a European perspective [31]
mining	Kathryn Moore	Mining of primary raw materials as the critical foundation of ‘sustainable’ metals: a wicked problem for technology innovation clusters [32]
recycling	Stefan Steinlechner	Supply security beyond mines and scrap recycling: valorization potential of metallurgical residues [33]
recycling	Rumana Hossain	Current recycling innovations to utilize e-waste in sustainable green metal manufacturing [34]

## 7. Conclusion

We introduce this issue as part of our own commitment to addressing the topic of ‘sustainable metals’ with humility and resolve. The fact that the problems have to date continued to worsen, despite the extravagant rhetoric of ‘sustainability’, is not an excuse to turn away or continue blindly following tracks that have failed to deliver. Instead, knowing that science and technology are integral to any solution, even if this is mainly through modifications to processes and materials that exist today, ensures we have a role. A significant and unfamiliar part of this role is to explain to others that there is no science-led solution that can scale rapidly enough to allow all of today’s familiar activities to continue without restraint and to apply the rigour of scientific methods to the process of rapid change.

## Data Availability

This article has no additional data.
